# Amiodarone Induced Epididymitis: A Case Report

**DOI:** 10.5812/ircmj.13929

**Published:** 2014-01-05

**Authors:** Tufan Cicek, Canan Cicek Demir, Gokcen Coban, Ali Coner

**Affiliations:** 1 Department of Urology, Baskent University Faculty of Medicine, Ankara, Turkey; 2 Department of Endocrinology and Metabolism, Baskent University Faculty of Medicine, Ankara, Turkey; 3 Department of Radiology, Baskent University Faculty of Medicine, Ankara, Turkey; 4 Department of Cardiology, Baskent University Faculty of Medicine, Ankara, Turkey

**Keywords:** Amiodarone, Epididymitis, Sterile

## Abstract

**Introduction:**

Amiodarone is an effective drug for life-threatening arrhythmias like recurrent ventricular fibrillation and atrial fibrillation. Amiodarone creates rarely genitourinary side effects are seen. These are epididymitis, testicular dysfunction and impotance. Amiodarone aggregates and triggers inflammation in the head of the epididym.

**Case report:**

We present the case of a patient who developed epididymitis after 17 months of amiodarone therapy, using a low dose (100 mg per day). Although cessation of medication or dose lowering was not performed, remission of the patient only by analgesics is a distinct case reported in urological literature.

**Conclusions:**

This case stresses the importance of considering an adverse effect of amiodarone treatment as a cause when making a differential diagnosis of epididymitis.

## 1. Introduction

Amiodarone is a class III antiarrhythmic and is often used in arrhythmias and atrial fibrillation. Amiodarone is lipid-soluble and extensively distributed in the body with a concentration of 300-fold the plasma level in many tissues, is associated with adverse reactions affecting the lung, skin, thyroid and nervous system (1). The accumulation of the amiodarone to the epididymis is related to the epididymal pain and swelling. Acute scrotum is a urological emergency therefore surgical exploration is needed in order to prevent needles surgical interventions scrotal pain and epididymitis should be considered in differential diagnosis with other causes of acute scrotum such as testicular torsion which is a “real” surgical emergency. Epididymitis is often associated with bacterial infections, rarely due to systemic tuberculosis and sexually transmitted infectious agents (2). Other uncommon causes are traumatic, obstructive and vasculitic diseases such as Behcet’s disease (1, 3). Non-infectious epididymitis must be diagnosed with a careful medical history to prevent unnecessary antibiotic usage.

## 2. Case Report

Here in we want to present an uncommon case of sterile epididymitis due to amiodarone therapy. A man aged 53 was consulted to our urology clinic in February 2012 (Konya/Turkey,) with left scrotal pain and edema ongoing for 3 days. He had no urethral discharge, disuria and suspicious sexual contact. He had hypertension and paroxysmal atrial fibrillation in his medical history. 

**Figure 1.**
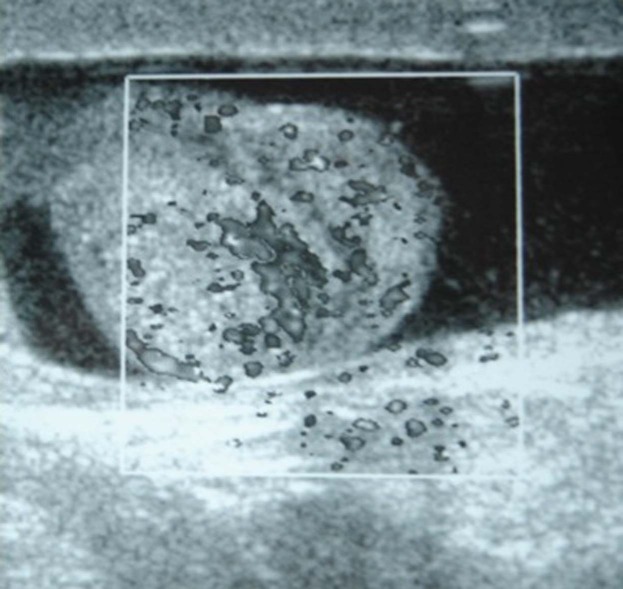
Color Doppler Ultrasonography Image Shows Increased Vascularization of the Left Epididymis.

He was on medication with Verapamil 1x240 mg, Irbesartan 1x150 mg, Acetylsalicylic acid 1x100 mg, Amiodarone 1x100 mg. He has been using Amiodarone at the same dosage for about 17 months. In his physical examination there was extreme sensitivity on his left epididym. No genital anatomical abnormality, no scrotal hyperemia and no fever were found. Also there was no testicular, penile or urethral mea abnormality. Scrotal doppler ultrasound imaging indicated increased vascularization in the left epididym that is suggestive for epididymal inflammation (Figure 1). 

**Figure 2.**
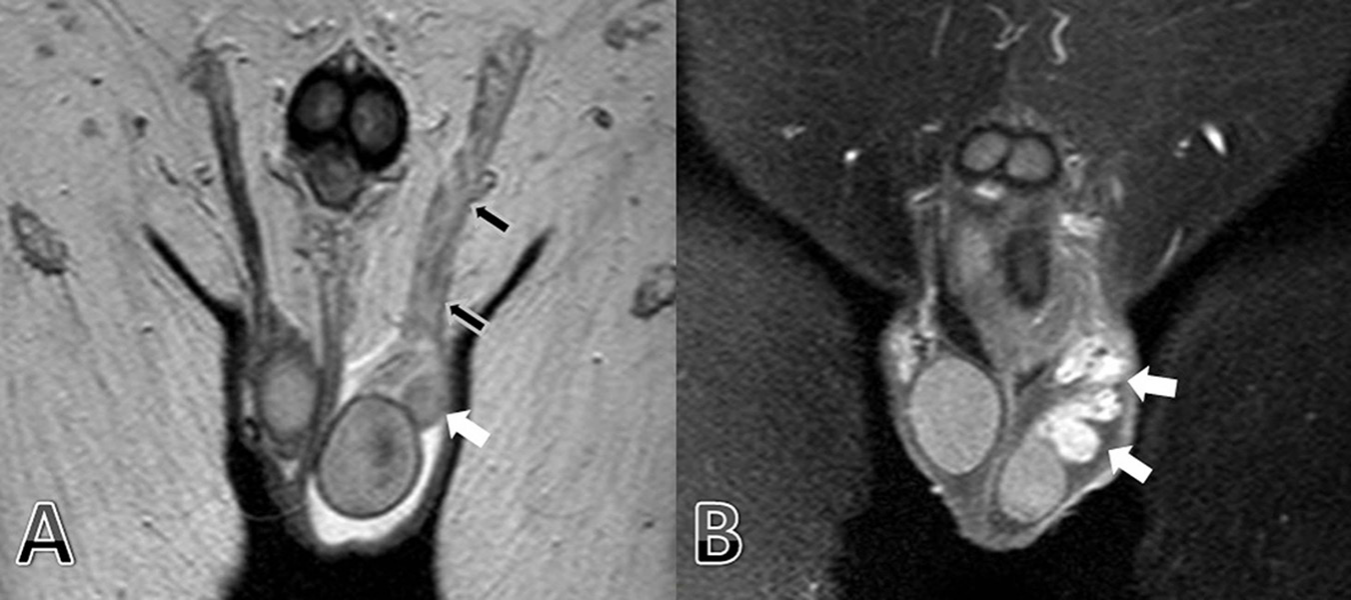
Coronal T2 Weighted (a) MR Imaging Shows Marked Edema of the Left Epididymis (Head-tail and Body, White Arrows) and Ductus Deferens (Black Arrows). After Contrast Media Administration on Coronal T1-weighted (b) MR Imaging Shows Marked Enhancement of the Left Epididymis (Head-Tail and Body, White Arrows).

Although complete blood count, C - reactive protein, urine analysis, prostate specific antigen, α-fetoprotein and β-Human chorionic gonadotropin was all in normal limits. Thyroid stimulating hormone level was 8.9 µIU/mL (0.4-4.4 µIU/mL) and antithyroid peroxidase antibody was negative. Thyroid ultrasound evaluation showed tissue heterogenity. 50mcg/day levothyroxine replacement therapy was started by endocrinology department. The patient was consulted to cardiology department to adjust medical treatment. Active cardiac complaint did not exist. Electrocardiography showed sinus rhythm with left bundle branch block. There was left ventricular concentric hypertrophy in his echocardiography.

Lower abdominal magnetic resonance was also taken for scrotal evaluation suitable with epididymal inflammation (Figure 2). 

Cardiologist was decided to continue Amiodarone treatment and decided to control the pain with analgesics. Upon received of the response to analgesic treatment in the first 2 days, patient was followed for 3 months of period. During this period of time he has continued Amiodarone medication at the same dosage. He defined that his scrotal pain was decreased little by little and he did not require any analgesic anymore. Doppler ultrasound imaging was repeated to control the vascularization of the epididyms, by same radiologist in order to avoid inter observer variability. This time there was an increased vascularity and edema in the right epididym and right sided moderate hydrocel. There was no increase in vascularity on the left epididym and also no hydrocel on the ultrasound check. Clinical follow up of the patient was performed by Tufan Cicek, MD and whole radiological evaluation was performed by Gokcen Coban, MD.

## 3. Discussion 

Epididymitis is usually occurs with gram negative bacterial agents and also mycobacterium tuberculosis and sexually transmitted diseases must be remembered in etiological causes (1). Also non-infectious causes such as post-traumatic epididymitis and obstructive pathology, Behcet’s disease and drug induced epididymitis may be encountered in differential diagnosis. The incidence of amiodarone epididymitis varies between 3% to 11% depending on the dosage and duration of usage (1) However, encountered only about 20-25 case reports in the evaluation of the literature (1, 4-6). Chronic epididymyalgia in the absence of fever and leucocytosis is the chief symptom of this clinical entity. As the pain and swelling subsided after amiodarone treatment was discontinued, amiodarone was the probable cause of the epididymitis.

Bilateral testicular involvement is generally present but usually pain in one side is more than the other masking epididymitis clinic and clinician may become suspicious falsely about anatomical abnormalities such torsion. Although epididymitis has been reported to occur in Amiodarone doses from 200 mg to 800 mg, it most often concerns doses more than 400 mg per day (4). Also beginning of symptoms of the epididymitis may vary from 4 to 71 months according to duration of the treatment (5). In adult population symptoms usually necessitate to stop the medication or to reduce the dosage of the drug however it is different from children patients (7). Regression of symptoms take a period of time from 10 days to 3 months in accordance with the slow elimination rate of drug varying from 25 days to 110 days (1). Exact etiology is unknown but possibilities are high concentration of Amiodarone and accumulation of its metabolite in epididymal tissue and histological changes such as focal epididymal fibrosis and lymphocytic infiltration. Anti-amiodarone antibodies may also play role in pathology (1).

In our case, 100 mg Amiodarone per day was enough to induce epididymalgia which is a very low dose considering the other cases since diagnosed. A close clinical follow-up and analgesics were proposed due to necessitate for the usage of Amiodarone for atrial fibrillation in the setting of ventricular hypertrophy. Within 3 months of symptom onset, the patient came to control and he indicated that the pain was disappeared and stated that he felt well despite the continuation of Amiodarone medication. He did not require any analgesia and symptoms had been decreased gradually. On the other hand radiological findings were well-matched with epididymitis while pain was totally resolute. Although our case was an interesting sample, despite the low dosage of Amiodarone onset epididymitis and resolution of pain with analgesia in the follow-up. Even though continuation of the drug is an interesting situation in discordance with Amiodarone induced epididymitis cases compared with previous literature. 
